# Development of a new prognostic model to predict pneumonia outcome using artificial intelligence-based chest radiograph results

**DOI:** 10.1038/s41598-024-65488-1

**Published:** 2024-06-22

**Authors:** Hyun Joo Shin, Eun Hye Lee, Kyunghwa Han, Leeha Ryu, Eun-Kyung Kim

**Affiliations:** 1 Department of Radiology, Research Institute of Radiological Science and Center for Clinical Imaging Data Science, Yongin Severance Hospital, Yonsei University College of Medicine, 363, Dongbaekjukjeon-daero, Giheung-gu, Yongin-si, Gyeonggi-do 16995 South Korea; 2 Division of Pulmonology, Allergy and Critical Care Medicine, Department of Internal Medicine, Yongin Severance Hospital, Yonsei University College of Medicine, 363, Dongbaekjukjeon-daero, Giheung-gu, Yongin-si, Gyeonggi-do 16995 South Korea; 3 Center for Digital Health, Yongin Severance Hospital, Yonsei University College of Medicine, 363, Dongbaekjukjeon-daero, Giheung-gu16995, Yongin-si, Gyeonggi-do South Korea; 4 Department of Radiology, Research Institute of Radiological Science and Center for Clinical Imaging Data Science, Severance Hospital, Yonsei University College of Medicine, 50-1 Yonsei-Ro, Seodaemun-Gu, Seoul, 03722 South Korea; 5 Department of Biostatistics and Computing, Yonsei University Graduate School, 50-1 Yonsei-Ro, Seodaemun-Gu, Seoul, 03722 South Korea

**Keywords:** Pneumonia, Artificial intelligence, Prognosis, Radiography, Mortality, Outcomes research, Infectious diseases, Respiratory tract diseases

## Abstract

This study aimed to develop a new simple and effective prognostic model using artificial intelligence (AI)-based chest radiograph (CXR) results to predict the outcomes of pneumonia. Patients aged > 18 years, admitted the treatment of pneumonia between March 2020 and August 2021 were included. We developed prognostic models, including an AI-based consolidation score in addition to the conventional CURB-65 (confusion, urea, respiratory rate, blood pressure, and age ≥ 65) and pneumonia severity index (PSI) for predicting pneumonia outcomes, defined as 30-day mortality during admission. A total of 489 patients, including 310 and 179 patients in training and test sets, were included. In the training set, the AI-based consolidation score on CXR was a significant variable for predicting the outcome (hazard ratio 1.016, 95% confidence interval [CI] 1.001–1.031). The model that combined CURB-65, initial O_2_ requirement, intubation, and the AI-based consolidation score showed a significantly high C-index of 0.692 (95% CI 0.628–0.757) compared to other models. In the test set, this model also demonstrated a significantly high C-index of 0.726 (95% CI 0.644–0.809) compared to the conventional CURB-65 and PSI (*p* < 0.001 and 0.017, respectively). Therefore, a new prognostic model incorporating AI-based CXR results along with traditional pneumonia severity score could be a simple and useful tool for predicting pneumonia outcomes in clinical practice.

## Introduction

Pneumonia is a common infectious disease and a leading cause of morbidity and mortality worldwide. In 2019, before the outbreak of covid infection, pneumonia was ranked the third leading cause of death after cancer and heart disease in Korea. According to the “Statistics on Causes of Death in 2019” released by the National Statistical Office of Korea, pneumonia accounted for 45.1 deaths per 100,000 population, which is a fourfold increase in mortality compared to the rate in 2008, which was 11.1 deaths per 100,000 population^1^.

Determining the severity of pneumonia is crucial because early risk prediction enables the determination of appropriate treatment policies and prediction of patient prognosis. To date, the most widely used severity scoring systems for predicting 30-day mortality in Community-acquired pneumonia (CAP) are the CURB-65 (confusion, urea, respiratory rate, blood pressure, and age ≥ 65) and pneumonia severity index (PSI)^2,3^. According to the 2019 ATS/IDSA guidelines, the classification of healthcare-associated pneumonia (HCAP), as distinct from CAP, is no longer recommended^4^. This change is due to the fact that patients with HCAP are not consistent risk factors for multi-drug resistant (MDR) pathogens, and there is no significant difference in the overall prognosis between CAP and HCAP. Several previous studies have also indicated that CURB-65 and PSI can be applied to HCAP, further demonstrating the similarities in clinical outcomes between HCAP and CAP^5–7^.

CURB-65 and PSI, however, are limited in that they do not adequately incorporate radiological indications, which are a crucial element of pneumonia. Recently, artificial intelligence (AI) has demonstrated to significantly improves the detection of pneumonia on chest radiographs (CXRs)^8,9^. Therefore, this retrospective study aimed to develop and evaluate the clinical usefulness of a prognostic prediction model that combined the CURB-65 and PSI to predict the prognosis of patients with pneumonia using AI-based CXR abnormality scores.

The purpose of this study was to develop a new, simple, and effective prognostic model incorporating AI-based CXR results to predict pneumonia outcomes.

## Results

### Patients

**Figure 1:**
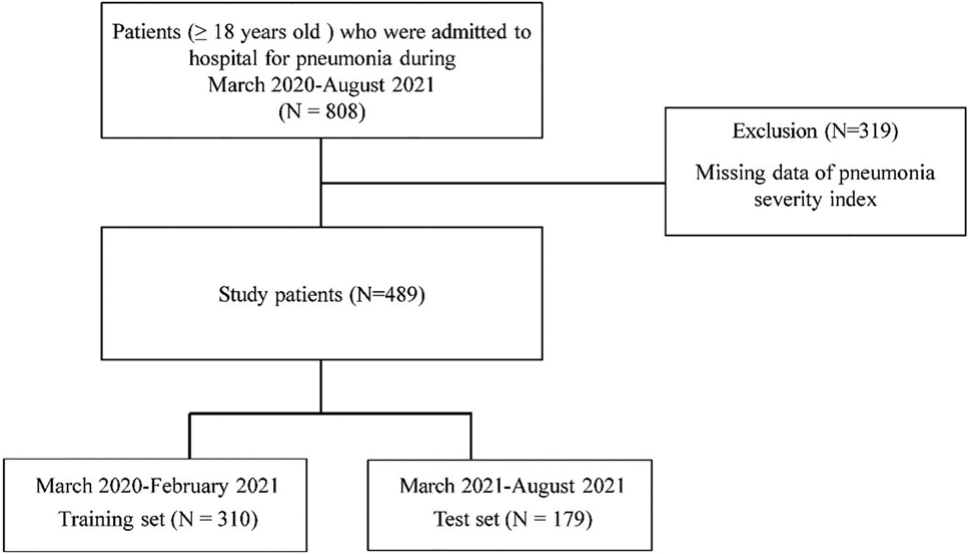
Flowchart of patient inclusion.

Among the 808 patients admitted with pneumonia, 319 were excluded because their PSI scores were not available. A total of 489 patients, including 310 and 179 in the training and test sets, respectively, were included in the final analysis. A flowchart of the inclusion and exclusion processes is presented in Fig. 1.

In the training set, age, CURB-65, PSI, initial O_2_ requirement, and AI-based consolidation score were significantly higher in the non-survivors. Other factors, such as being a nursing home resident, do not resuscitate (DNR) status, intensive care unit (ICU) admission, and intubation, showed no significant difference in the training set. Baseline characteristics of patients in the training and test sets are presented in Table 1.

**Table 1:** Baseline characteristics of patients in the training and test sets.

Groups	Training set			Test set		
Variables	Survivals (n = 250)	30-day mortalities (n = 60)	*P*-value	Survivals (n = 144)	30-day mortalities (n = 35)	*P*-value
Age (year), (mean (SD))	77.09 (12.00)	82.35 (7.81)	0.001	74.23 (15.31)	77.74 (10.55)	0.201
Gender, male, n (%)	154 (61.6)	42 (70.0)	0.288	91 (63.2)	24 (68.6)	0.690
Smoking, n (%)						
Never	191 (76.4)	51 (85.0)	0.203	88 (61.1)	21 (60.0)	> 0.999
Ever	59 (23.6)	e		56 (38.9)	14 (40.0)	
CURB-65 (%)						
0	23 (9.2)	0 (0.0)	0.007	18 (12.5)	2 (5.7)	0.056
1	71 (28.4)	17 (28.3)		56 (38.9)	12 (34.3)	
2	122 (48.8)	26 (43.3)		59 (41.0)	13 (37.1)	
≥ 3	34 (13.6)	17 (28.3)		11 (7.6)	8 (22.9)	
PSI (mean (SD))	123.43 (33.97)	130.60 (26.45)	0.128	114.85 (37.17)	129.31 (31.58)	0.035
I–II (< 70)	12 (4.8)	1 (1.7)	0.037	18 (12.5)	0 (0.0)	0.149
III (71–90)	28 (11.2)	1 (1.7)		20 (13.9)	4 (11.4)	
IV (91–130)	114 (45.6)	26 (43.3)		52 (36.1)	15 (42.9)	
V (> 130)	96 (38.4)	32 (53.3)		54 (37.5)	16 (45.7)	
Initial O_2_ requirement (L/min, %) *						
0 (room air)	58 (23.2)	2 (3.3)	< 0.001	32 (22.2)	0 (0.0)	0.016
1–2	76 (30.4)	17 (28.3)		29 (20.1)	6 (17.1)	
3–6	56 (22.4)	9 (15.0)		42 (29.2)	17 (48.6)	
> 6, < 15	15 (6.0)	9 (15.0)		12 (8.3)	5 (14.3)	
≥ 15*	45 (18.0)	23 (38.3)		29 (20.1)	7 (20.0)	
Nursing home resident, n (%)	92 (36.8)	22 (36.7)	> 0.999	34 (23.6)	10 (28.6)	0.695
DNR, n (%)	109 (43.6)	31 (51.7)	0.326	16 (11.1)	25 (71.4)	< 0.001
ICU admission, n (%)	58 (23.2)	21 (35.0)	0.086	20 (13.9)	17 (48.6)	< 0.001
Intubation, n (%)	38 (15.2)	16 (26.7)	0.056	19 (13.2)	17 (48.6)	< 0.001
Length of hospital stay, day, median (IQR)	12.00 [7.00, 19.00]	7.00 [2.00, 16.00]	< 0.001	12.00 [7.00, 19.25]	13.00 [4.50, 21.00]	0.845
Consolidation score on CXR, % (mean (SD))	81.00 (24.79)	88.49 (19.30)	0.030	72.46 (29.49)	88.24 (22.68)	0.004

*Include high flow O2 therapyValues are presented as median with interquartile ranges or number with percentage.

CURB, confusion, urea, respiratory rate, blood pressure, and age ≥ 65, PSI, pneumonia severity index; DNR, do not resuscitate; ICU, intensive care unit; CXR, chest radiographs; SD, standard deviation; IQR, interquartile range.

### Cox regression analysis for the new prognostic models in the training set

In the univariate Cox regression analysis, age, CURB-65, initial O_2_ requirement, intubation, and consolidation score on CXR were significant variables for predicting pneumonia mortality (Table 2). Therefore, we formed six prognostic models including initial O_2_ requirement, intubation, and consolidation score in addition to the conventional CURB-65 and PSI for predicting pneumonia outcomes as follows; Model A: CURB-65; Model B: PSI; Model C: CURB-65, initial O_2_ requirement, and consolidation score; Model D: CURB-65, initial O_2_ requirement, intubation, and consolidation score; Model E: PSI, consolidation score; and Model F: PSI, intubation, and consolidation score. Other significant variables, such as age for all and initial O_2_ requirement for PSI, were not included in the models because they were already incorporated into the scoring systems themselves.

**Table 2:** Univariate Cox regression analysis of variables in the training set.

Variable	Coefficient	HR (95% CI)	*P*-value
Age	0.042 (0.015, 0.068)	1.043 (1.015, 1.071)	**0.002**
CURB-65	0.468 (0.177, 0.758)	1.596 (1.194, 2.135)	**0.002**
PSI	0.006 (− 0.001, 0.013)	1.006 (0.999, 1.013)	0.11
Initial O_2_ requirement	0.042 (0.019, 0.065)	1.043 (1.019, 1.067)	**< 0.001**
DNR	0.305 (− 0.202, 0.811)	1.356 (0.817, 2.251)	0.238
ICU admission	0.506 (− 0.024, 1.037)	1.659 (0.976, 2.821)	0.061
Intubation	0.651 (0.079, 1.223)	1.918 (1.082, 3.399)	**0.026**
Length of hospital stay	− 0.006 (− 0.023, 0.012)	0.994 (0.977, 1.012)	0.516
Consolidation score on CXR	0.016 (0.001, 0.03)	1.016 (1.001, 1.031)	**0.038**

CURB-65, confusion, urea, respiratory rate, blood pressure, and age ≥ 65; PSI, pneumonia severity index; DNR, do not resuscitate; ICU, intensive care unit; CXR, chest radiographs; HR, hazard ratio; CI, confidence interval.

Significant values are in bold.

The results of the multivariate Cox regression analysis using these models are presented in Table 3. Model C showed a significantly higher C-index compared to Model A (0.685 vs. 0.595, *p* = 0.003), Model B (0.685 vs. 0.583, *p* = 0.011), and Model E (0.685 vs. 0.615, *p* = 0.037). Additionally, Model D showed a significantly higher C-index compared to Model A (0.692 vs. 0.595, *p* = 0.002), Model B (0.692 vs. 0.583, *p* = 0.005), Model E (0.692 vs. 0.615, *p* = 0.023), and Model F (0.692 vs. 0.624, *p* = 0.028). Therefore, we chose Model D for its high predictive value of pneumonia outcomes.

**Table 3:** Multivariate Cox regression analysis and new prognostic models in the training set.

Models	Variables	Coefficient	HR (95% CI)	*P*-value	C-index (95% CI)	*P*-value for comparison of C-index				
						Comparison with A	Comparison with B	Comparison with C	Comparison with D	Comparison with E
A	CURB-65	0.468 (0.177, 0.758)	1.596 (1.194, 2.135)	0.002	0.595 (0.528, 0.662)					
B	PSI	0.006 (− 0.001, 0.013)	1.006 (0.999, 1.013)	0.11	0.583 (0.52, 0.646)	0.764				
C	CURB-65	0.469 (0.174, 0.023)	1.598 (1.19, 1.024)	0.002	0.685 (0.619, 0.751)	**0.003**	**0.011**			
	Initial O_2_ requirement	0.048 (0.174, 0.023)	1.05 (1.19, 1.024)	< 0.001						
	Consolidation score	0.013 (0.174, 0.023)	1.013 (1.19, 1.024)	e						
D	CURB-65	0.434 (0.134, 0.023)	1.544 (1.144, 1.023)	0.005	0.692 (0.628, 0.757)	**0.002**	**0.005**	0.517		
	Initial O_2_ requirement	0.048 (0.134, 0.023)	1.049 (1.144, 1.023)	< 0.001						
	Intubation	0.419 (0.134, 0.023)	1.52 (1.144, 1.023)	0.162						
	Consolidation score	0.012 (0.134, 0.023)	1.012 (1.144, 1.023)	0.107						
E	PSI score	0.005 (− 0.002, 0)	1.005 (0.998, 1)	0.154	0.615 (0.546, 0.685)	0.657	0.36	**0.037**	**0.023**	
	Consolidation score	0.015 (− 0.002, 0)	1.015 (0.998, 1)	0.046						
F	PSI	0.005 (− 0.002, − 0.001)	1.005 (0.998, 0.999)	0.158	0.624 (0.554, 0.695)	0.53	0.283	0.089	**0.028**	0.635
	Intubation	0.574 (− 0.002, − 0.001)	1.776 (0.998, 0.999)	0.0504						
	Consolidation score	0.014 (− 0.002, − 0.001)	1.014 (0.998, 0.999)	0.067						

CURB-65, confusion, urea, respiratory rate, blood pressure, and age ≥ 65; PSI, pneumonia severity index; HR, hazard ratio; CI, confidence interval.

Significant values are in bold.

### Validation in the test set and simplified prognostic model

**Figure 2:**
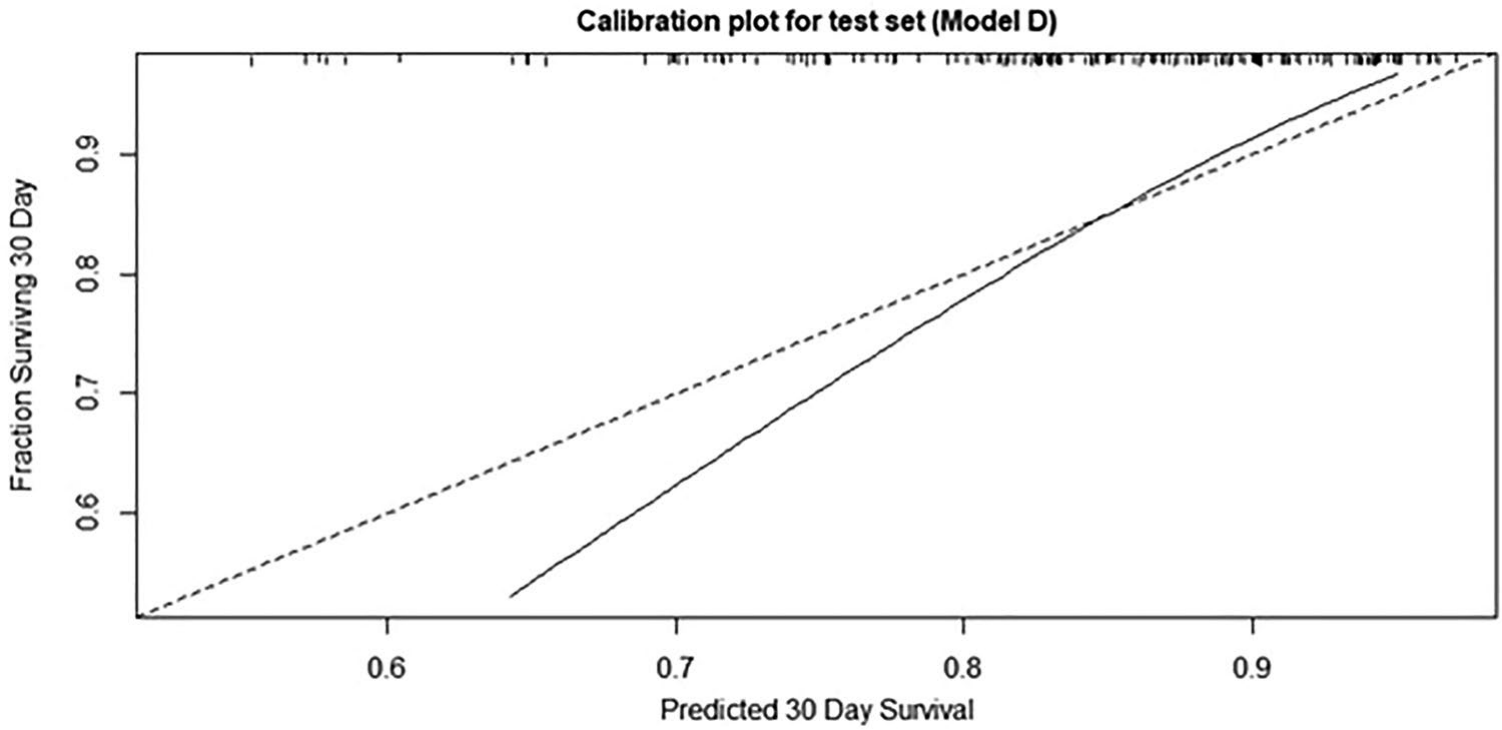
The calibration plot of model D. The calibration plot of model D for test set showed a relatively good fit to the 45-degree line.

Therefore, we applied Model D in the test set to demonstrate its prognostic value for time-independent external validation. In the test set, Model D showed a C-index of 0.726 (95% confidence interval [CI] 0.644–0.809), which was significantly higher compared to that of Model A–C. The C-index of Model D was higher compared to Model E, while there was no statistical significance (0.726 vs. 0.696, *p* = 0.461), and it also showed no significant difference compared to Model F (0.726 vs. 0.759, *p* = 0.313) (Table 4). The IBS of model D was 0.088 and the calibration plot showed a relatively good fit to the 45-degree line (Fig. 2).

**Table 4:** Comparison of C-index of new prognostic models in the test set.

Models	C-index (95% CI)	*P*-value for comparison of C-index				
		Comparison with A	Comparison with B	Comparison with C	Comparison with D	Comparison with E
A (CURB-65)	0.599 (0.504, 0.694)					
B (PSI)	0.593 (0.505, 0.681)	0.92				
C (CURB-65 + Initial O_2_ requirement + Consolidation score)	0.694 (0.603, 0.784)	**0.002**	0.064			
D (CURB-65 + Initial O_2_ requirement + Intubation + Consolidation score)	0.726 (0.644, 0.809)	**<0.001**	**0.017**	**0.042**		
E (PSI + consolidation score)	0.696 (0.614, 0.778)	0.069	**0.003**	0.947	0.461	
F (PSI + Intubation + Consolidation score)	0.759 (0.685, 0.834)	**0.004**	**0.001**	0.102	0.318	**0.031**

CURB-65, confusion, urea, respiratory rate, blood pressure, and age ≥ 65; PSI, pneumonia severity index; CI, confidence interval.

Significant values are in bold.

## Discussion

In the present study, we showed that a new prognostic score composed of AI-based CXR results improved the prediction of pneumonia prognosis when combined with the previously known pneumonia severity score. In predicting the prognosis of pneumonia, the patient’s vital signs, age, underlying disease, extent of pneumonia invasion, and radiologic features are expected to affect the prognosis; however, the degree of radiologic involvement has not previously been quantified on CXRs, making it difficult to include them as prognostic predictors. However, AI technology has provided a commercial tool that quantitatively shows abnormality scores representing the probability of containing lesions on the image and has made it possible to combine the results with existing pneumonia severity measurement tools^10,11^. Our findings showed that the power of predicting the patient’s prognosis and mortality increased when the consolidation score on CXRs presented by AI was combined with the patient’s clinical characteristics.

Recently, new risk stratification methods have been introduced for predicting pneumonia outcomes, especially for coronavirus disease 2019 (COVID-19), with or without AI^12–15^. The use of AI on images for creating new prognostic markers has attracted more attention. Further, in recent studies, AI-based quantification of increased opacity areas on CXRs has been shown to be an independent predictor of adverse outcomes in patients with COVID-19^16,17^. Jiao et al. extracted deep learning-based features from the CXRs of patients with COVID-19 and validated a new model combining image and clinical data for predicting disease severity^18^. They demonstrated that AI-based medical image results could enhance the prognostic value of clinical data in determining disease progression. In addition to CXRs, AI-based quantification of chest computed tomography has been used as a predictive indicator for patients with COVID-19^19^.

Besides COVID-19, only one study has reported an AI-based method for the analysis of CXRs to predict 30 day-mortality in CAP^8^. It demonstrated that a deep learning-based model incorporated with the PSI showed the best prognostic performance in patients with CAP. However, the study only included patients with CAP and the authors developed their own deep-learning model for scoring areas of increased opacities on CXRs. Our study used commercially available AI software that is known to have an excellent diagnostic performance^20–22^. This software offers individual abnormality scores of eight lesions, including consolidation separately, which could be a more objective method than combining increased opacity areas on CXRs^9,23,24^.

In this study, Model D, incorporating AI-based CXR results along with CURB-65, initial O_2_ requirement, and intubation, demonstrated enhanced predictive power in the training set and validation in the test set, while its integration with the PSI showed minimal impact. One possible reason for this discrepancy could be that the PSI itself incorporates pleural effusion, one of the CXR-based imaging findings, which may diminish the additional effect of consolidation compared to CURB-65. Moreover, since the PSI comprises a complex set of variables, it may already demonstrate better prognostic accuracy than CURB-65. However, conversely, given that CURB-65 is more clinically utilized than the complex PSI, adding imaging metrics to CURB-65 may result in a more clinically practical prognostic score. Thus, this study’s advantage lies in providing a simpler prognostic score with higher clinical utility. Therefore, combining AI-based CXR results to the simple CURB-65 had additive and practical value in clinical use for predicting pneumonia outcomes and had the potential to be widely utilized clinically.

This study has several limitations. Firstly, this was a retrospective study conducted at a single center, which may introduce biases in data collection and affect the generalizability of the findings due to a discrete sample size. Additionally, the inclusion of a significant proportion of nursing home residents and DNR patients, about one-third of the participants, could have confounded the results. Moreover, the predictive value of the PSI was found to be inferior to that of the CURB-65, and the integration of AI-based CXR results with PSI did not significantly enhance predictive outcomes. Secondly, the use of only one commercial software for CXR analysis may further limit the generalizability of the results. Although it was capable of detecting various lesions, only consolidation was included as it is the most representative feature of pneumonia. To address these issues, we underwent external validation of the prognostic value of the models using a time-independent test set. Third, there may be issues regarding whether other lung abnormalities, such as pleural effusion or atelectasis, were included or affected in the AI analysis, and whether the projection view of CXR or the use of portable equipment could have influenced the accuracy of AI diagnosis. The commercial AI software used in this study does not restrict image analysis based on the differentiation between anteroposterior and posteroanterior views or portable equipment. Moreover, since consolidation is a prominent imaging finding for pneumonia, only representative imaging findings were added to the analysis. Whether concurrent lung lesions affect the diagnosis of AI is currently an area of interest for AI researchers. This is also an important topic in AI research, and it should be addressed with focus in well-designed studies. The research team plans to conduct further studies in the future to explore this issue, as it requires more validation. Our model benefits from using a commercially available AI software and proposed prognostic models that could be utilized and reproduced in other institutes, offering a comparison with other research-based AI algorithms developed specifically for dedicated hospitals.

In conclusion, our study demonstrates that a new prognostic model incorporating AI-based CXR result, along with traditional pneumonia severity scores, could provide a simple and effective method for predicting pneumonia outcomes. Further multicenter large-scale studies are necessary to confirm the predictive power of these prognostic models.

## Methods

### Patients and clinical data

The Institutional Review Board of our hospital approved this retrospective study (IRB no. 9-2021-0028) and the requirement for informed consent was waived. The study was conducted according to Strengthening the Reporting of Observational Studies in Epidemiology [STROBE] guidelines. Patients aged > 18 years, who were admitted to our hospital for the treatment of pneumonia between March 2020 and August 2021 were included. For the development of a new prognostic score for the prediction of death as an outcome of pneumonia, patients admitted from March 2020 to February 2021 were included in the training set, and those admitted from March 2021 to August 2021 were included in the test set. We excluded patients who did not have AI-based CXR results or data on the CURB-65 scores, PSI, or initial O_2_ requirement. We reviewed patients’ electronic medical records to retrieve all individual risk factors comprising the CURB-65 scores and PSI based on patient demographics and baseline clinical data^25^. The CURB-65 scores and PSI were calculated using the collected data (Table S1–2 in Supplementary file 1)^3,26^.

### Analysis of AI-based CXR results

**Figure 3:**
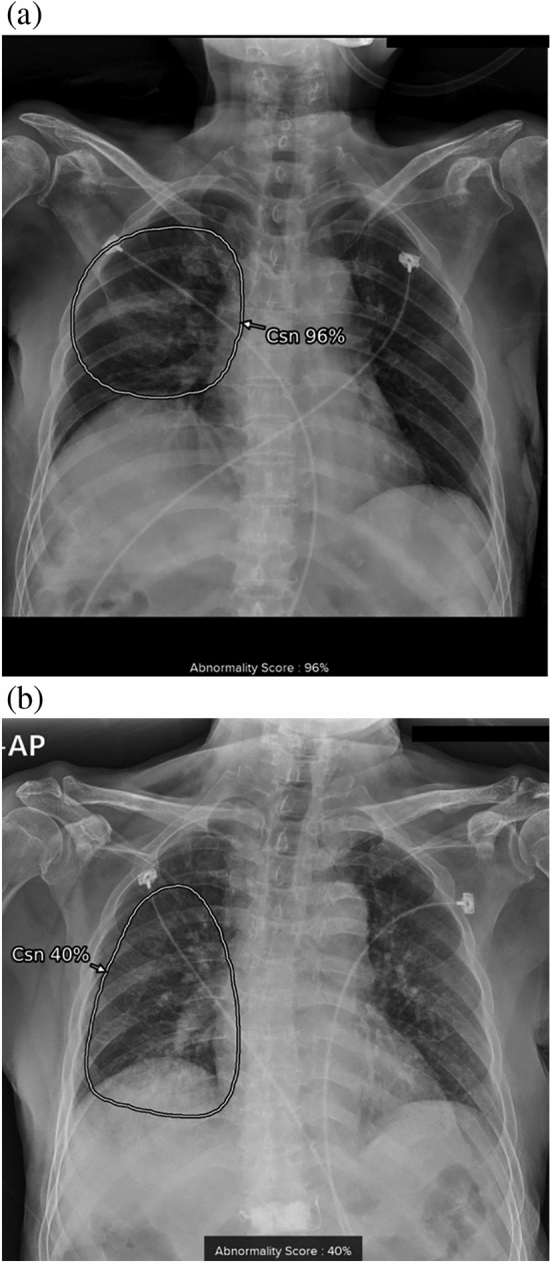
Example cases of patients with pneumonia. (**a**) A patient with a CURB-65 of 2 and PSI of 97 had an initial O_2_ requirement of 6L, no intubation, and an AI-based consolidation score of 96% on the initial CXR. This patient died during admission for pneumonia treatment. (**b**) A patient with a CURB-65 of 1 and PSI of 117 had an initial O_2_ requirement of 2L, no intubation, and a consolidation score of 40% on the initial CXR. This patient recovered and was discharged after treatment. CURB-65, confusion, urea, respiratory rate, blood pressure, and age ≥ 65; CXR, chest radiograph; PSI, pneumonia severity index; Csn, consolidation.

We assessed the abnormality score for each CXR taken at the time of admission due to pneumonia to develop a new simple and effective prognostic score containing AI-based CXR results. In our hospital, commercially available AI-based lesion detection software (Lunit INSIGHT CXR, version 2 and 3, Lunit Inc., Korea) has been integrated for all CXRs since March 2020. This software can detect consolidation regardless of the software version with high diagnostic performance and can be used to calculate an abnormality score for a detected lesion and display a contour map on a secondary capture image of the CXR when the abnormality score is > 15% (Fig. 3)^9,20,27^. Doctors can refer to the AI results by scrolling down the original CXR on a picture archiving communication system (PACS). Therefore, we extracted the abnormality score of consolidation on the first CXR acquired upon admission of the patients. Among the detectable lesions, we selected consolidation because this is a representative imaging feature of CXR in patients with pneumonia. The abnormality score represented probability that the CXR would contain abnormal lesions and ranged from 0 to 100%. We used the abnormality score for consolidation on CXR as a continuous variable, reflecting the imaging severity of pneumonia in each patient.

### Development and validation of new prognostic scoring system for predicting outcomes

Pneumonia outcomes were determined by evaluating factors affecting 30-day mortality during admission. In the training set, significant factors for predicting outcome were determined using the variables of the CURB-65 score, PSI, initial O_2_ requirement, intubation, and consolidation score on the CXR. Several prognostic models using these variables were analyzed in the training set. External validation was performed using test set for temporal validation of the models using the significant models in the training set. Afterward, simplified new scoring system was presented to calculate point values for the selected model^28^.

### Statistical analysis

We used the R program (version 4.2.3, Foundation for Statistical Computing, Vienna, Austria, package: survival, rms, compareC, pec) for the statistical analysis. Patient demographics were compared using a *t* test for continuous variables after the normality test and chi-square test for categorical variables. Significant variables for predicting death during admission as a pneumonia outcome were determined by univariate Cox regression analysis in the training set. Using the significant variables, several prognostic models were developed and a multivariate Cox regression model was performed in the training set. The C-index was assessed using the 1000 times bootstrapping method and the differences between groups were evaluated^29^. About the significant models, we evaluated the C-index in the test set for temporal validation^28^. The integrated brier score (IBS) and calibration plot were presented to show the performance and calibration of the selected simplified prognostic model^28^. Statistical significance was set than 0.05, and corrected *p*-values < 0.0125 were considered significant using the Bonferroni method to minimize the chance of type 1 error for the multiple comparison of the C-index in the test set.

### Ethical approval

This study was performed in accordance with the amended Declaration of Helsinki and was approved by the institutional review board (IRB) of Yongin Severance hospital (approval no: 9-2021-0028). The need for informed consent was waived by the IRB of Youngin Severance Hospital due to the retrospective nature of the study.

### Supplementary Information

Supplementary Tables.

Supplementary Information 2.

## Data Availability

All datasets for this study are contained in Supplementary file 2.

## Abbreviations

AI: Artificial intelligence
CXR: Chest radiograph
CURB-65: Confusion, urea, respiratory rate, blood pressure, and age ≥ 65
PSI: Pneumonia severity score
HR: Hazard ratio
CI: Confidence interval
