# Supplementary material for: Development of a new prognostic model to predict pneumonia outcome using artificial intelligence-based chest radiograph results
Source: Sci Rep. 2024 Jun 22;14:14415. doi: 10.1038/s41598-024-65488-1 (PMC11193777; doi:10.1038/s41598-024-65488-1)
Supplement: Supplementary file 1 — Supplementary Tables. [file 41598_2024_65488_MOESM1_ESM.docx]

**Supplementary file 1.**

**Table S1. The features used and method of calculation of the CURB-65 score.**

| **Features** | **Criteria** | **Points** |
| --- | --- | --- |
| Confusion | Presence | +1 |
| Blood urea | > 19 mg/dL (> 7 mmol/L) | +1 |
| Respiratory rate | ≥ 30/min | +1 |
| Blood pressure | Systolic blood pressure < 90 mmHg or diastolic blood pressure ≤ 60 mmHg | +1 |
| Age | ≥ 65 years | +1 |

Abbreviations: CURB-65; confusion, urea, respiratory rate, blood pressure, and age ≥65.

**Table S2. The features used and method of calculation of PSI.**

| **Features** | **Criteria** | **Points** |
| --- | --- | --- |
| Age, sex | Male, female | Male: age  Female: age -10 |
| Nursing home resident | Yes | +10 |
| Neoplastic disease | Present | +30 |
| History of liver, congestive heart, cerebrovascular, or renal diseases | Present | +20 (liver) or +10 (others), for each |
| Altered mental status | Present | +20 |
| Respiratory rate | ≥ 30/min | +20 |
| Systolic blood pressure | < 90 mmHg | +20 |
| Body temperature | <35 or >39.9 °C | +15 |
| Pulse rate | ≥ 125/min | +10 |
| Arterial pH | < 7.35 | +30 |
| Blood urea nitrogen | ≥ 30 mg/dL (9 mmol/L) | +20 |
| Sodium | < 130 mmol/L | +20 |
| Glucose | ≥ 250 mg/dL (14 mmol/L) | +10 |
| Hematocrit | < 30% | +10 |
| Partial pressure of arterial O_2_ | < 60 mmHg | +10 |
| Pleural effusion | Present | +10 |

Abbreviations: PSI; pneumonia severity index.
